# Osteopaths Must Leave Kentucky

**Published:** 1900-01

**Authors:** 


### OSTEOPATHS MUST LEAVE KENTUCKY.

KENTUCKY has once more rendered a service to humanity and the cause of legitimate medicine by pushing to successful conclusion an action in the courts relating to osteopathy. The facts are briefly as follows: Harry Nelson, an osteopath, applied for an injunction against the Kentucky state board of health, to stay proceedings against him instituted to prevent his practising medicine in Kentucky. He claimed to be a graduate of the osteopathic institution of Kirksville, Mo., which he alleged was a reputable school.

The state board of health having investigated the Kirksville institution by a special committee sent there for that purpose, denied that it was a reputable school of medicine. It appears that the authorities in control at Kirksville have erected a good building, and expended large sums of money to foist their deceptive scheme upon an unwary public.

Judge Sterling B. Toney, before whom the case was tried, held that there were but two points at issue; first, the constitutionality of the act to protect citizens from empiricism; and, second, whether the Kirksville school was a reputable institution. He said the word reputable was not used here in its ordinary sense as when applied to individual character, but that it included a broader meaning as to the standing of the school among educational institutions, and whether it could register with the state boards as a legally qualified medical school.

Judge Toney's opinion embraced a full review of the case and the essential points may be summarised as follows:

> As to the first question, Judge Toney held that the act was constitutional, quoting a number of state and federal decisions in support of his position. As to the second, whether or not the Missouri institution was a reputable medical college, one of good standing, he ruled that this was a question of fact to be decided by the State Board of Health; that that board had instituted a careful investigation of this matter, appointing a special committee to do this, and that as a result of this investigation it had decided that the Missouri School was not a reputable medical college, although legally chartered under the laws of that state. The board's decision on this question of fact Judge Toney held was final and conclusive, and was not a matter for judge or jury to pass upon. The injunction was refused and petition dismissed.

The state board of health regards the decision as effective in removing the last obstacle to freeing the state from the only remaining relic of quackery within its borders. It is to be regretted that the lawyers and courts of New York have not been as efficient in this work as they have been in Kentucky. Here it is difficult to inspire a lawyer with courage and enthusiasm sufficient to bring action, or to secure conviction when issue is made, or the courts to sentence when conviction is obtained. In Kentucky the bench and bar and all people of education are loyal to legitimate medicine, and desirous of maintaining its purity and high standards.

